# Opportunistic Bacteria of Grapevine Crown Galls Are Equipped with the Genomic Repertoire for Opine Utilization

**DOI:** 10.1093/gbe/evad228

**Published:** 2023-12-12

**Authors:** Hanna Faist, Markus J Ankenbrand, Wiebke Sickel, Ute Hentschel, Alexander Keller, Rosalia Deeken

**Affiliations:** Center for Health & Bioresources, Bioresources Unit, AIT Austrian Institute of Technology GmbH, Tulln 3430, Austria; Julius-von-Sachs Institute for Biological Sciences, Molecular Plant Physiology and Biophysics, University of Würzburg, Würzburg 97082, Germany; Faculty of Biology, Center for Computational and Theoretical Biology, University of Würzburg, Würzburg 97074, Germany; Institute of Biodiversity, Thuenen-Institute of Biodiversity, Braunschweig 38116, Germany; RD3 Marine Ecology, RU Marine Symbioses, GEOMAR Helmholtz Centre for Ocean Research Kiel, Kiel 24105, Germany; Sektion Biologie, Christian-Albrechts University of Kiel, Kiel 24105, Germany; Cellular and Organismic Networks, Faculty of Biology, Ludwig-Maximilians-Universität München, Planegg-Martinsried 82152, Germany; Julius-von-Sachs Institute for Biological Sciences, Molecular Plant Physiology and Biophysics, University of Würzburg, Würzburg 97082, Germany

**Keywords:** *Vitis vinifera*, bacterial community, *Agrobacterium*, *Allorhizobium vitis*, Ti plasmids, de novo sequenced genomes

## Abstract

Young grapevines (*Vitis vinifera*) suffer and eventually can die from the crown gall disease caused by the plant pathogen *Allorhizobium vitis* (*Rhizobiaceae*). Virulent members of *A. vitis* harbor a tumor-inducing plasmid and induce formation of crown galls due to the oncogenes encoded on the transfer DNA. The expression of oncogenes in transformed host cells induces unregulated cell proliferation and metabolic and physiological changes. The crown gall produces opines uncommon to plants, which provide an important nutrient source for *A. vitis* harboring opine catabolism enzymes. Crown galls host a distinct bacterial community, and the mechanisms establishing a crown gall–specific bacterial community are currently unknown. Thus, we were interested in whether genes homologous to those of the tumor-inducing plasmid coexist in the genomes of the microbial species coexisting in crown galls. We isolated 8 bacterial strains from grapevine crown galls, sequenced their genomes, and tested their virulence and opine utilization ability in bioassays. In addition, the 8 genome sequences were compared with 34 published bacterial genomes, including closely related plant-associated bacteria not from crown galls. Homologous genes for virulence and opine anabolism were only present in the virulent Rhizobiaceae. In contrast, homologs of the opine catabolism genes were present in all strains including the nonvirulent members of the Rhizobiaceae and non-Rhizobiaceae. Gene neighborhood and sequence identity of the opine degradation cluster of virulent and nonvirulent strains together with the results of the opine utilization assay support the important role of opine utilization for cocolonization in crown galls, thereby shaping the crown gall community.

**Significance**

Virulent *Allorhizobium vitis* causes crown galls on grapevines which reduce plant vigor and yield and cannot be cured. Nonvirulent agrobacteria have been used as biocontrol agents to reduce the virulence potential within a crown gall and disease symptoms. We wanted to know if and how in nature this biocontrol concept is accomplished. We found virulent *Allorhizobium* along with nonvirulent *Agrobacterium* or *Pseudomonas* in the same tumors. Both harbored the catabolism genes in their genomes and metabolized the *quorum sensing* molecule opine. Thus, in nature, it seems common that virulent and nonvirulent species coexist in a crown gall and that the avirulent members control the virulence potential of the crown gall community by reducing the opine levels.

## Introduction

*Allorhizobium vitis* (*Rhizobiaceae*), former *Agrobacterium vitis* or *Agrobacterium tumefaciens* biovar 3, is the causal pathogen of grapevine crown galls (CGs) which hamper plant growth and yield (Schroth et al. 1988; Ferreira et al. 1992). Overall, the family *Rhizobiaceae* contains both virulent and nonvirulent species (Bien et al. 1990; Chandrasekaran et al. 2019), and *Rhizobiaceae* are associated with different grapevine tissues (Burr and Katz 1983). Virulence is encoded by the bacterial tumor-inducing plasmid (Ti plasmid; Wikipedia contributors 2023) that consists of the transfer DNA (T-DNA), the virulence gene operon (including *vir* genes), genes encoding opine utilization enzymes, as well as a bacterial backbone region that regulates replication and conjugation of the Ti plasmid (Chen and Xie 2011; Gordon and Christie 2014). The Vir proteins guide the transfer of the T-DNA into the plant nucleus and enable integration into the plant genome. The transformed plant cells express T-DNA-encoded genes, consequently leading to the production of plant growth hormones and opines (Gelvin 2010). Uncontrolled production of the plant hormones auxin and cytokinin causes plant cell proliferation and thereby tumorous growth, also referred to as CGs (Gohlke and Deeken 2014; Escobar et al. 2001; Klee et al. 1984). CGs do not only offer space for virulent *Rhizobiaceae* but also for a specific bacterial community which is distinct and differs from the community of a normal wound callus of the graft union (Faist et al. 2016). Moreover, a recent study on the bacterial composition of 73 CGs has shown that at least 3 non-*A. vitis* groups coexist in CGs (Gan et al. 2019)

Opines produced by CG cells are a source of nitrogen and carbon for *A. vitis*, and the capacity to utilize opines provides a fitness advantage over non–opine-utilizing bacteria (Lang et al. 2017). For virulent *Rhizobiaceae*, opines not only represent a nutrient source but also are involved in *quorum sensing* that regulates e.g. Ti plasmid conjugation and its distribution between bacteria (Ellis et al. 1982; Wetzel et al. 2014). Different virulent *Rhizobiaceae* transfer different opine biosynthesis genes into the plant genome. Various opines are known (Moore et al. 1997; Dessaux et al. 1998; Chilton et al. 2001), and in CGs, nopaline, octopine/cucumopine, and/or vitopine/heliopine have been found of which the latter opine type exclusively occurs in grapevine CGs (Szegedi et al. 1988; Szegedi 2003). It has been postulated that the vitopine/heliopine-type pTi's of *A. vitis* represent a distinct group of Ti plasmids (Szegedi et al. 1996). Indeed, the bacterial catabolism genes on the Ti plasmid correspond to these opine types. Nevertheless, as opines are produced by T-DNA transformed plant cells, they are a public good in a CG (Platt et al. 2012). Consequently, other bacteria of the CG community may also utilize opines as a nutrient source, promoting their enrichment in CGs. For example, some *Pseudomonas* strains isolated from CGs can utilize opines (Bergeron et al. 1990; Moore et al. 1997).

In our study, we provide the draft genome sequences of 3 virulent *A. vitis* isolates and 5 nonvirulent bacterial isolates of grapevine CG communities (3 *Rhizobiaceae*, 1 *Pseudomonas*, and 1 *Rahnella*) and analyzed these strains for virulence. In octopine and nopaline utilization bioassays, we tested growth of the 8 grapevine CG isolates. In addition, 34 published genomes of plant-associated bacteria were included in our analyses to identify orthologous genes of the Ti plasmids. In our genome analysis, we focused on the distribution of genes involved in virulence, Ti plasmid conjugation (quorum sensing), and opine metabolism in the CG bacterial communities.

## Results

### Physical Characteristics of the 8 Genomes

The 8 de novo sequenced bacterial genomes belonged to isolates from 5 different grapevine CGs harvested in the region Franconia, Bavaria, and Germany (supplementary table S1, Supplementary Material online). According to EZBioCloud (Yoon, et al. 2017), the isolates CG1 to CG6 belonged to the *Rhizobiaceae* family, CG7 was identified as *Pseudomonas* sp., and CG8 was identified as *Rahnella* sp. (supplementary table S1, Supplementary Material online). The screening for amplicon sequencing variants (ASVs) of the data set published by Faist et al. (2016) revealed that the V4 regions of the 16S rRNA sequences from the isolates CG1 to CG3 and CG7 to CG8 were significantly enriched in CGs compared with healthy graft unions (Table 1), whereas ASVs of CG4 to CG6 showed no significant enrichment (Faist et al. 2016).

**Table 1:** Information about the bacterial genomes analyzed in this study (1 to 3 and 7 to 11) and the known reference sequences (4 to 6 and 12 to 16) used for comparison

	Name^a^ (database accession number and/or reference)	Selection criteria	Predicted CDS	tRNAs, rRNAs	Essential genes (completeness), missing genes
	**Virulent Rhizobiaceae**				
1.	*A. vitis* CG1, this study	*Rhizobiaceae*, enrichment	5,727	56, 12	106 (99.1%), TIGR01030 (rpmH)
2.	*A. vitis* CG2, this study	*Rhizobiaceae*, enrichment	5,051	49, 3	106 (99.1%), TIGR01030 (rpmH)
3.	*A. vitis* CG3, this study	*Rhizobiaceae*, enrichment	5,550	50, 3	106 (99.1%), TIGR01030 (rpmH)
4.	*A. vitis* S4 (Slater et al. 2009, CP000637.1)	Weisberg et al.	5,729^b^	55, 12^b^	…
5.	*A. fabrum* C58 (Goodner et al. 2001, Wood et al. 2001, SAMN02603108)	Weisberg et al.	5,253^b^	53, 12^b^	…
6.	*A. rhizogenes* K84 (SAMN02602977)	Weisberg et al.	6,836^b^	52, 9^b^	…
7.	*A. vitis* NCPPB3554 (K309) (SAMN04223557)	Weisberg et al.	na	na	…
8.	*A. rhizogenes* CG101/95 (SAMN14165425)	Weisberg et al.	6,185^b^	49, 3^b^	…
9.	*A. rhizogenes* CM79/95 (SAMN14165429)	Weisberg et al.	6,931^b^	48, 3^b^	…
10.	*A. vitis* BM37/95 (SAMN14165430)	Weisberg et al.	5,398^b^	49, 2^b^	…
11.	*A. vitis* P86/93 (SAMN14165436)	Weisberg et al.	5,392^b^	50, 3^b^	…
12.	*A. vitis* T268/95 (SAMN14165437)	Weisberg et al.	5,497^b^	49, 3^b^	…
13.	*A. vitis* T60/94 (SAMN14165442)	Weisberg et al.	5,509^b^	50, 3^b^	…
14.	*A. rhizogenes* U167/95 (SAMN14165443)	Weisberg et al.	6,205^b^	47, 2^b^	…
15.	*A. rhizogenes* D1/94 (SAMN14165450)	Weisberg et al.	6,692^b^	48, 3^b^	…
16.	*A. vitis* T267/94 (SAMN14165455)	Weisberg et al.	5,458^b^	50, 3^b^	…
17.	*A. vitis* T393/94 (SAMN14165459)	Weisberg et al.	5,069^b^	50, 2^b^	…
18.	*A. vitis* V80/94 (SAMN14165463)	Weisberg et al.	5,359^b^	49, 2^b^	…
19.	*A. vitis* AV25/95 (SAMN14165468)	Weisberg et al.	5,255^b^	50, 2^b^	…
20.	*A. rhizogenes* CM65/95 (SAMN14165470)	Weisberg et al.	6,554^b^	48, 3^b^	…
21.	*A. tumefaciens* CG53/95 (SAMN14165473)	Weisberg et al.	5,469^b^	51, 3^b^	…
22.	*A. rhizogenes* T155/95 (SAMN14165487)	Weisberg et al.	6,240^b^	47, 3^b^	…
23.	*A. rhizogenes* CM80/95 (SAMN14165490)	Weisberg et al.	6,939^b^	48, 2^b^	…
24.	*A. vitis* CG412 (SAMN14165504)	Weisberg et al.	4,706^b^	50, 6^b^	…
25.	*A. vitis* CG678 (SAMN14165505)	Weisberg et al.	5,332^b^	45, 3^b^	…
26.	*A. vitis* CG78 (SAMN14165506)	Weisberg et al.	5,332^b^	45, 3^b^	…
27.	*A. vitis* F2/5 (SAMN14165507)	Weisberg et al.	5,251^b^	48, 3^b^	…
	**Nonvirulent Rhizobiaceae**				
28.	*A. divergens* CG4, this study	*Rhizobiaceae*	5,470	48, 3	106 (99.1%), TIGR01030 (rpmH)
29.	*Rhizobiaceae* sp. CG5, this study	*Rhizobiaceae*	6,251	46, 3	105 (98.1%), TIGR00631 (uvrB), TIGR01030 (rpmH)
30.	*A. rosae* CG6, this study	*Rhizobiaceae*	5,569	51, 6	107 (100%), TIGR01030 (rpmH)
	**Other proteobacteria**				
31.	*Pseudomonas* sp. CG7, this study	Enrichment	6,002	69, 4	103 (96.3%), TIGR00810 (secG), TIGR02432 (tilS), TIGR03594 (engA), TIGR01030 (rpmH)
32.	*Rahnella* sp. CG8, this study	Enrichment	5,099	71, 4	No missing genes
33.	*Pseudomonas cerasi* (Kałużna et al. 2016, SAMEA3894894)	Enrichment	5,725^b^	63, 15^b^	…
34.	*R. aquatilis* HX2 (Guo et al. 2012, SAMN02603118)	Biocontrol	5,127^b^	76, 22^b^	…
35.	*Sphingomonas* sp. Leaf230 (SAMN04151690)	Reduction	3,613^b^	51, 3^b^	…
36.	*Pseudomonas congelans* H346-M (SAMN15924736)	Enrichment	4,944^b^	57, 3^b^	…
37.	*Pseudomonas savastanoi pv. glycinea* (SAMN03976351)	Enrichment	na	na	…
38.	*Pseudomonas avellanae* (SAMN05861163)	Enrichment	5,782^b^	56, 4^b^	…
39.	*P.* sp. *18058* (SAMEA6372291)	Enrichment	na	na	…
40.	*Pseudomonas koreensis* Cl12 (SAMN06018396)	Enrichment	5,792^b^	70, 15^b^	…
	**Actinobacteria**				
41.	*Curtobacterium flaccumfaciens* MCBA15_005 (SAMN05736482)	Reduction	3,465^b^	45, 4^b^	…
42.	*C.* sp. strain 6 (Bulgari et al. 2014, SAMN02709057)	Endophytic	na	na	…

^a^Species names as originally published. The selection criteria for the sequences were (i) “enrichment” or “reduction” of the 16S rRNA V4 amplicon sequences in CGs in springtime (Faist et al. 2016), (ii) member of the *Rhizobiaceae* family, (iii) part of the agrobacterial virulence plasmid study (Weisberg et al. 2020), (iv) endophytic in grapevine graft unions without CG disease, and (v) biocontrol for CG disease. Annotation statistics for genomes from this study or according to NCBI assembly report (genome annotation data, if available, indicated with ^b^). na, not available; CDS, coding sequences.

The general features of the assembled 8 draft genomes are summarized in supplementary table S2, Supplementary Material online. The length of the smallest contigs, which accounted for 90% of the genome (N90 index), ranged from 17.6 kb (CG7, *Pseudomonas*) to 204 kb (CG6, *Rhizobiaceae* sp.). The *A. vitis* draft genomes (CG1 to CG3) shared a GC content of around 57%, while it varied between the other *Rhizobiaceae* isolates (CG4 to CG7, 55% to 61.5%). *Rahnella* (CG8) possessed the lowest GC content with 52.3%. Mapping the raw reads to the draft genomes resulted in ∼98% alignment rates for CG1, CG2, and CG4 to CG6 and about ∼85% to 87% for the isolates CG3, CG7, and CG8.

Inoculation assays demonstrated that the 3 *A. vitis* isolates (CG1 to CG3) induced CG development on stems of in vitro cultivated grapevine plantlets but not the other 3 Rhizobiaceae isolates (CG4 to CG6; supplementary fig. S1, Supplementary Material online). Like the latter 3, *Pseudomonas* (CG7) and *Rahnella* (CG8) did not induce CG development even in stems of the test plants *Arabidopsis thaliana* and *Nicotiana benthamiana*. Thus, the isolates CG1 to CG3 were confirmed as virulent, while CG4 to CG8 were nonvirulent.

### Relationship between Isolates and Reference Genomes

Information about the selection criteria of the de novo sequenced draft genomes (CG1 to CG8) and the reference sequences of known plant-associated bacteria used for phylogenetic relationship analysis are summarized in Table 1. The numbers of predicted coding sequences (CDS) and tRNAs are similar among the isolates, while the number of rRNAs varies, most likely due to the highly challenging assembly of frequently duplicated genes in draft genomes. At least 103 out of 107 essential genes were found in all de novo sequenced isolates, indicating a completeness of the draft genomes of at least 96%.

**Fig. 1.**
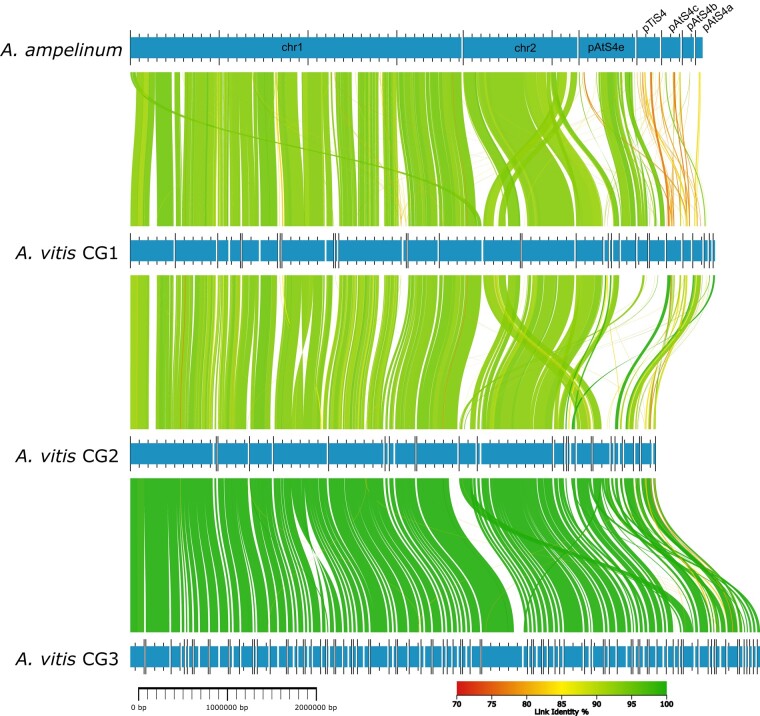
DNA alignment of *A. vitis* genomes. Horizontal bars indicate the chromosomes (chr1, chr2) and plasmids (pATS4e, pTiS4, pTiS4a-c) of *A. ampelinum* and the contigs of the *A. vitis* CG1 to CG3 genome sequences. Homologous regions between genomes are connected via lines of different colors. A color gradient (70% to 100%) according to the similarity (link identity %) is provided.

**Fig. 2.**
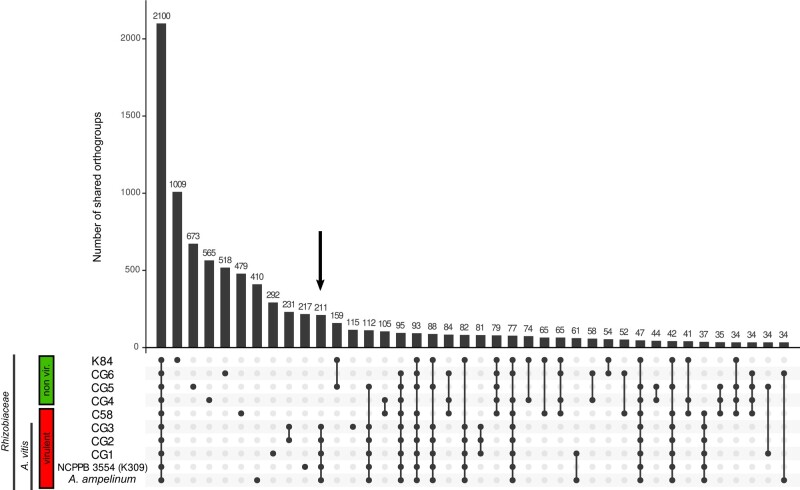
Shared and unique OGs encoded by the sequences of the *Rhizobiaceae* genomes (CG1 to CG6) and the references *A. ampelinum*, *A. vitis* NCPPB3554 (K309), *A. fabrum* C58, and *A. rhizogenes* K84. Each column represents the number of orthologous genes only shared by the genomes (dots below each bar). The boxes indicate virulent bacteria and nonvirulent bacteria. Less than 34 shared protein families are not shown.

The *Rhizobiaceae* family encompasses virulent and nonvirulent members including the monophyletic groups of *Agrobacterium* and *Allorhizobium* (Gan and Savka 2018). The results of a phylogenetic tree generated on the basis of 107 housekeeping gene sequences revealed that the 2 nonvirulent *Rhizobiaceae* isolates CG4 (*Agrobacterium divergens*) as well as CG6 (*Agrobacterium rosae*) belong to the clade of *Agrobacterium*, while CG5 is related to *Allorhizobium* (supplementary fig. S2, Supplementary Material online). The 3 virulentisolates CG1 to CG3 formed a clade also with the genus *Allorhizobium* of which CG2 to CG3 belonged to the branch of *A. vitis*. A whole genome alignment using the program AliTV performed with *Allorhizobium ampelinum* and the draft genomes of the isolates CG1 to GC3 revealed that the 2 chromosomes and the pATS4e plasmid express high homology (Fig. 1, green connecting lines, >85% homology), while the pTiS4 plasmid possessed much lower homology (orange to yellow connecting lines, <5% homology). The close relationship between the virulent *A. vitis* genomes was confirmed by the number of protein families shared (Fig. 2). CG1, CG2, and CG3 exclusively shared 211 protein families with *A. ampelinum* and *A. vitis* NCPPB3554 (K309) (Fig. 2, arrow).

### Relationships between Isolates and Reference Ti Plasmids

**Fig. 3.**
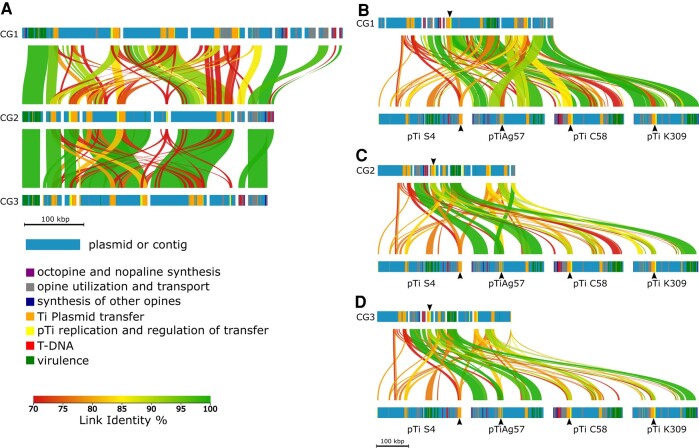
DNA alignments of Ti plasmid regions of the de novo sequenced isolates CG1 to CG3 and 3 reference pTis. Alignment of (A) CG1, CG2, and CG3 and (B to D) of potential Ti plasmids from CG1 to CG3 to the reference Ti plasmids of *A. ampelinum* (vitopine/heliopine type), *A. fabrum* Ag57 (octopine/cucumopine type), *A. fabrum* C58 (nopaline type), and *A. vitis* NCPPB3554 (pTiK309) (octopine/cucumopine type). Horizontal bars represent contigs of the potential Ti plasmids and reference pTis. The position of predicted protein functions is marked by different colors. Homologous regions between the contigs are connected via vertical-colored lines, and the color gradient shows % similarity (link identity %). Black arrow heads indicate the regions for Ti plasmid transfer (orange bars) and pTi plasmid replication (yellow bars) with highest homology to the reference plasmids.

The relationship between Ti plasmids was investigated by aligning the potential pTi sequences of our de novo sequenced strains with each other and with the reference sequences of pTiS4 (vitopine/heliopine type), pTiAg57 (octopine/cucumopine type), pTiC58 (nopaline type), and pTiK309 (octopine type). The potential Ti plasmids of the sequenced isolates CG1, CG2, and CG3 are more homologous to each other than to the reference pTi sequences. Among the 3, CG2 and CG3 show a higher degree of homology to each other than to CG1 (Fig. 3A). The alignments of CG1 to CG3 to the reference pTis revealed that large parts of the CG1 contigs have strong homology (more than 99% identity) to long regions of pTiAg57 and almost the complete pTiK309 sequence (Fig. 3B, dark green connecting lines). In particular, the *vir* regions (Fig. 3B to D, green bars) of the de novo sequenced *Allorhizobium* isolates (CG1 to CG3) are highly homologous (100%) to the *vir* regions of pTiAg57 and pTiK309 but much less to pTiS4 (orange connecting lines) and pTiC58 (red connecting lines). Furthermore, other contigs of CG1 to CG3 encoding opine synthesis (Fig. 3 purple bars), opine utilization and transport (Fig. 3, gray bars), as well as plasmid replication and transfer (Fig. 3, yellow bars) match to a high degree to pTiAg57.

### Prediction of Ti Plasmid Encoding Protein Families

**Fig. 4.**
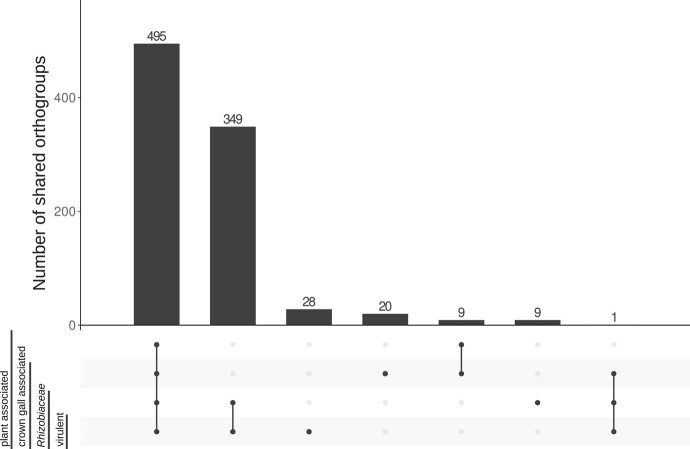
Shared and unique protein families among the selected genomes. These are grouped into (i) plant associated, (ii) CG associated, (iii) Rhizobiaceae, and (iv) virulent. The 4 groups consist of the following members: (i) *P. cerasi*, *R. aquatilis*, *S.* sp. Leaf230, *Curtobacterium strain 6*, and *C. flaccumfaciens*, (ii) *Pseudomonas* CG7 and *Rahnella* CG8, (iii) nonvirulent *Rhizobiaceae CG4* to *CG6*, and (iv) *A. fabrum C58*, *A. ampelinum*, and the virulent *A. vitis* isolates C*G1* to *CG3*. A protein family is part of a group if it occurs in all members (dots). A protein family is not part of a group if it occurs in none of the members. Protein families that occur in some representatives of a group are discarded.

To functionally describe the de novo sequenced genomes of the 8 CG isolates, we compared the predicted protein sequences with those of the reference strains. The predicted proteins were clustered into orthologous groups (OGs) (in short: orthogroups) by a set of amino acid sequences derived from a single common ancestor sequence for all the isolates (Emms and Kelly 2015). In total, 21,431 unique predicted orthogroups were identified, and for a selection of 15 plant-associated bacteria, including the 8 CG isolates and 7 of the reference genomes, 495 orthogroups were shared by all the isolates (Fig. 4). The genomes of the *Rhizobiaceae* had 349 additional unique orthogroups in common, and the taxonomically heterogeneous genomes of CG-associated bacteria shared only a single unique orthogroup. However, the subgroup of virulent bacterial genomes exclusively shared 28 orthogroups with each other.

The proteins known to be encoded by the Ti plasmids (pTiC58, pTiS4, pTiK309, pTiAg57) of the reference bacteria *A. tumefaciens* C58, *A. ampelinum*, *A. vitis* NCPPB3554, and *Agrobacterium fabrum* Ag57, respectively, are listed in Table 2 and assigned to all of our isolates CG1 to CG8. The predicted protein families functioning in virulence and CG induction were present in the genomes of the 3 virulent isolates CG1 to CG3 but largely absent in the genomes of the nonvirulent bacteria (Table 2A). An exception was VirG which is part of the sensory response system and therefore found in all analyzed genomes in substantial numbers. Proteins related to plasmid replication and transfer (Tra, Trb, RepABC) were not only present in the virulent CG1 to CG3 but also in the nonvirulent isolates CG4, CG5, and CG6, while they were missing in *Pseudomonas* sp. (CG7) and *Rahnella* sp. (CG8) (Table 2B). Protein families predicted to be associated with opine utilization such as opine catabolism (OoxA/B, NoxA/B, VoxA/B) occurred in all analyzed genomes (Table 2C) and were not unique to the virulent bacteria in contrast to those for opine anabolism (Vis, Cus, Ocs, Acs, Acsx). Proteins functioning in opine transport (e.g. AccC-E and OccPch, NocP) were found in large numbers. Taken together, virulence, opine degradation, and T-DNA-encoded protein families were predominantly found in the genomes of the virulent *A. vitis* isolates. Those predicted to transfer and replicate Ti plasmids were present in all *Rhizobiaceae* isolates, and proteins for opine transport and catabolism occurred in all listed plant-associated bacteria.

**Table 2:** Number of predicted protein families encoded by the Ti plasmids of the reference bacterial strains *A. fabrum* C58 (pTiC85) and LBA649 (pTiAg57), *A. ampelinum* (pTiS4), *A. vitis* NCPPB3554 (K309), and *A. fabrum* Ag57 (pTiAg57) as well as the de novo sequenced draft genomes of the CG-associated isolates CG1 to CG8. GABA sensing genes are encoded on pAtC58 (NC_003064). Representative names of the predicted protein families are listed in the first column. Gene counts are from whole genome, not only from plasmids, except for pTiAg57 where only the plasmid sequence is known

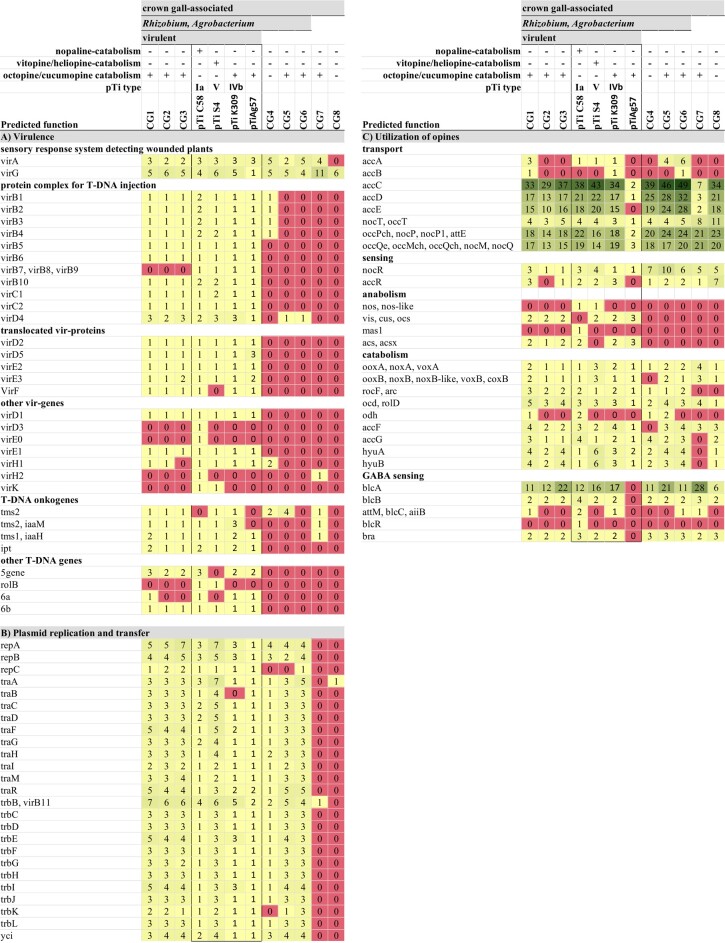

### Genetic Basis for Opine Utilization Match In Vitro Opine Bioassay

**Fig. 5.**
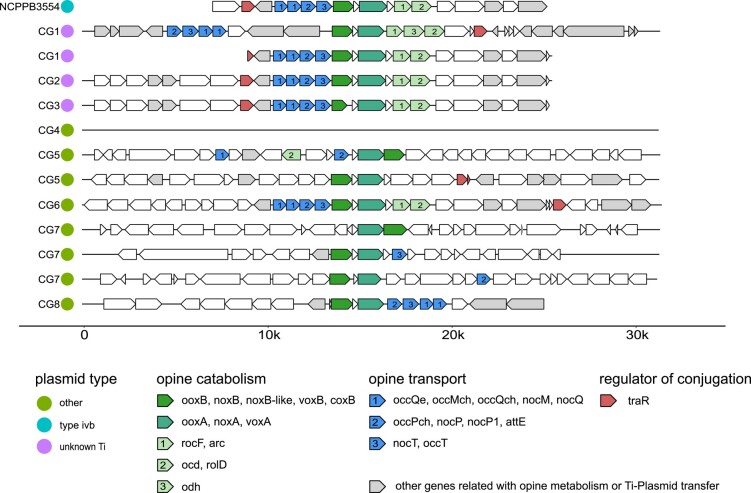
Regions in the de novo sequenced genomes of the CG1 to CG8 isolates from grapevine CGs that contain orthologs to both ooxA and ooxB, compared with the octopine catabolism cluster of pAtK309. The horizontal black lines represent a part of a contig centered around the ooxA/B, noxA/B, and voxA/B genes. Arrows symbolize the orientation of coding regions including known annotations of Prokka. Green colors symbolize opine catabolism; blue, opine transport; red, regulator of plasmid conjugation; and gray, other genes related with opine metabolism or Ti plasmid transfer. The number within the arrows specifies the gene function within a cluster.

All CG-associated isolates, except CG4, contained representatives of the well-described Nox/Oox/Vox protein families, essential for opine degradation. Figure 5 visualizes the order and orientation of the predicted proteins in the vicinity of the subunits A and B of Oox/Nox/Vox (green and turquoise arrows) in the de novo sequenced genomes and the reference *A. vitis* NCPPB3554 (K309). The fragments of the virulent *A. vitis* isolates CG1 to CG3 showed exactly the same structure as the reference. This included the NoxA/B, OoxA/B, and VoxA/B protein families (Fig. 5, green and turquoise arrows), other protein families related to opine catabolism (Fig. 5, pale green arrows), and those for opine transport (Fig. 5, blue arrows) upstream of OoxB/NoxB/VoxB (Fig. 5, green arrows). Isolate CG1 harbored a second cluster with additional genes between the transport protein family and OoxB/NoxB/VoxB. Growth assays with the virulent CG1 to CG3 isolates in liquid AB salt medium supplemented with opines as sole carbon and nitrogen source, confirmed the function of the opine catabolism clusters (supplementary table S3, Supplementary Material online). The isolates utilized octopine better than nopaline pointing to the presence of octopine catabolism gene clusters in their genomes.

**Fig. 6.**
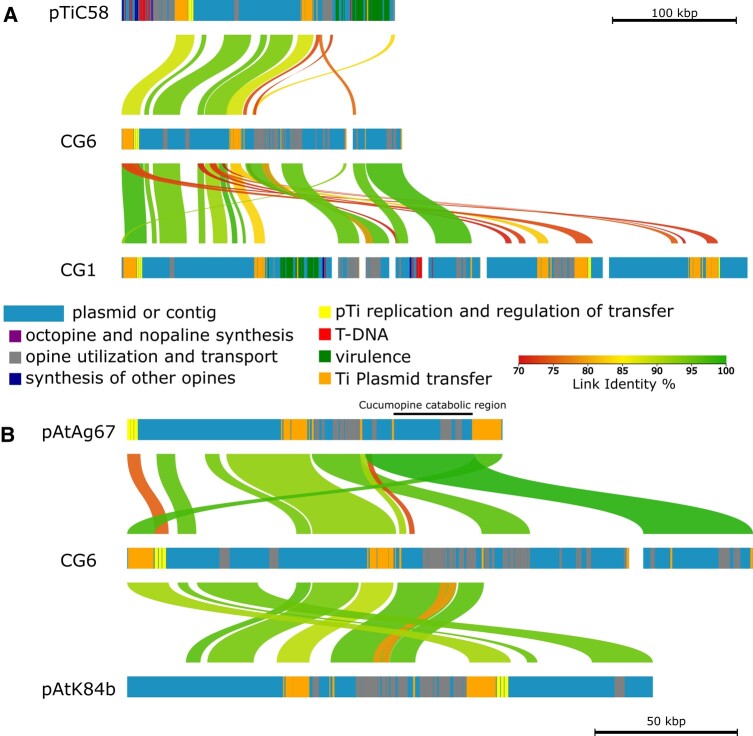
DNA alignment of putative plasmid sequences with reference plasmids. Horizontal bars represent the contigs and encoded protein families colored according to their function. Homologous regions between the sequences are connected via lines in a color gradient corresponding to % similarity (link identity %). A) Alignment of pTi regions from the reference strain *A. fabrum C58* and putative plasmid sequences of 2 isolates, the virulent *A. vitis* CG1, and nonvirulent *A. rosae* CG6. B) Alignment of the opine-catabolic plasmid regions from the virulent reference *A. fabrum* Ag57 (pAtAg67), the nonvirulent agrocinopine/nopaline-type plasmid of *A. rhizogenes K84* (pAtK84b), and putative plasmid sequences of the nonvirulent *A. rosae* CG6. The cucumopine-catabolic region of pAtAg67, according to Hooykaas et al. (2022), is indicated with a black bar.

The 3 nonvirulent *Rhizobiaceae* isolates CG4, CG5, and CG6 showed a different behavior concerning opine utilization. *Agrobacterium divergens* (CG4) did not grow, but *Rhizobiaceae* sp. (CG5) grew well and *A. rosae* (CG6) weakly in liquid AB salt medium supplemented with octopine (supplementary table S3, Supplementary Material online). Accordingly, the opine degradation region of CG4 lacked all substantial components (Fig. 5, black line), while the 2 clusters of CG5 harbored the essential genes but in a different order as compared with the virulent isolates CG1 to CG3, except of the Nox/Oox/Vox proteins (Fig. 5). CG6 possessed the same opine cluster structure as the *A. vitis* NCPPB3554 (K309) reference strain and the virulent isolates *A. vitis* CG1 to CG3 but grew only weekly in opine containing liquid medium (supplementary table S3, Supplementary Material online). A closer inspection of CG6 revealed additional predicted protein sequences that were homologous to those of the isolate CG1 and the reference plasmid pTiC58 of *A. fabrum* C58 (Fig. 6A). In the reference plasmid, the homologs were related to replication and regulation of transfer (yellow bars), Ti plasmid transfer (orange bars), and opine utilization and transport (gray bars). Regions related to virulence (green bars) and the T-DNA (red bars) were not detected in the isolate CG6 genome but in the isolate CG1. This underlined the finding that CG6 was unable to induce CGs (supplementary fig. S1, Supplementary Material online) but able to metabolize octopine, although not very well (supplementary table S3, Supplementary Material online). Therefore, we compared the opine utilization and transport regions of CG6 also with the nonvirulent agrocinopine/nopaline-type plasmid pAtK84b of *Agrobacterium rhizogenes K84* and the octopine/cucumopine-catabolic *A. fabrum* plasmid pAtAg67 of the narrow host range *A. fabrum* strain Ag57. We found higher sequence identity between those than between CG6 and the Ti plasmid of C58 (Fig. 6B, green connecting lines between gray bars). Particularly, 1 contig of CG6 matched the cucumopine-catabolic region of pAtAg67 with 99.5% identity. Thus, it is likely that the opine utilization and transport region of CG6 belongs to an opine-catabolic plasmid, most likely a cucumopine type, rather than to a Ti plasmid.

The draft genome of the *Pseudomonas* isolate CG7 contained 3 regions with homology to the OoxA/B, NoxA/B, and VoxA/B protein families; none of them has all the genes like the reference (NCPPB3554; Fig. 5). However, the CG7 isolate grew well in liquid AB salt medium containing octopine like *A. rosae* CG6 and despite the differences in the structure of the opine clusters to those of the virulent isolates CG1 to CG3 (supplementary table S3, Supplementary Material online). In the *Rahnella* CG8 genome, the DNA sequences encoding the OoxB/NoxB/VoxB protein family were also present in the classical orientation upstream of OoxA/NoxA/VoxA (Fig. 5). However, in contrast to the opine utilization clusters of CG1 to CG3, the opine transporter genes (Fig. 5, blue arrows) in CG8 were downstream of OoxA/B, NoxA/B, VoxA/B and the isolate was not able to metabolize opines (supplementary table S3, Supplementary Material online). Taken together, our study reveals that the nonvirulent CG-associated bacteria *A. rosae* CG6 and *Pseudomonas* CG7 possess functional opine transport and catabolism sequence regions.

## Discussion

Virulent *A. vitis* strains generate a new ecological niche in plants by transferring a T-DNA into the plant genome that leads to neoplastic growth of grapevine tissue so-called CGs. In contrast to normal stem tissues, CGs form a sink tissue characterized by a hypoxic environment and accumulation of sugars, amino acids, and opines (Deeken, et al. 2006) which are exclusively produced by T-DNA harboring host cells. In addition to the pathogen, a seasonally stable microbiota resides in the nutrient-rich CG environment (Faist et al. 2016).

This study aims to unravel whether homologs of the protein-encoding genes typically located on the Ti plasmid of virulent agrobacteria are also found in nonvirulent bacterial members of the CG bacterial community. To identify these genes in CG-associated bacteria, we included genomes of non–tumor-associated plant bacteria in our analysis. Thereby, we focused on the functions: virulence, plasmid replication, and transfer (conjugation), as well as opine utilization summarized in Table 2. The distribution of the underlying genes may allow us to draw conclusions regarding the importance of their role in CG ecology.

### Virulence Is Restricted to *A. vitis* in the CG Community

Three out of the 8 de novo sequenced bacterial isolates (CG1, CG2, CG3) analyzed in this study belonged to the taxonomic group of *Allorhizobium*. They were isolated from different grapevine CGs, and in an infection assay, they induced neoplastic growth on in vitro cultivated grapevines. The sequences comprising the chromosomes of CG1 to CG3 were largely homologous to those of the well-characterized strain *A. ampelinum* (Fig. 1). Concerning the Ti plasmid sequences, the 3 genomes showed a higher homology to each other than to the reference Ti plasmids of *A. ampelinum* (vitopine/heliopine type), *A. fabrum* Ag57 (octopine/cucumopine type), *A. fabrum* C58 (nopaline type), and *A. vitis* pTiK309 (octopine type Fig. 3). The Ti plasmid pTiAg57 was more similar to the putative plasmid contigs of CG2 and CG3, than any of the other reference plasmids (pTiS4, pTiC58, pTiK309). These findings support the idea of (i) an independent propagation of the chromosomes and Ti plasmids, as previously suggested (Slater et al. 2009), and (ii) a faster ecological specification of plasmids encoding virulence in contrast to chromosomes encoding mainly housekeeping functions (Weisberg et al. 2020). Both factors must be considered for the development and interpretation of diagnostic tests targeting the CG disease.

Essential for CG development are the virulence regions located on the Ti plasmid that are involved in the process of T-DNA transformation and have previously been summarized for *A. fabrum C58* (Pitzschke and Hirt 2010). Our *A. vitis* isolates CG1 to CG3 shared similar predicted protein sequences associated with virulence of the Ti plasmids of *A. fabrum* C58 (pTiC58), *A. ampelinum* (pTiS4), *A. vitis* NCPPB3554 (pTiK309), and *A. fabrum* Ag57 (pTiAg57). Among those are the proteins known to be important for (i) recognition of the host cell and induction of Vir gene expression (VirA, VirG), (ii) the type IV secretion system (T4SS: VirB2, VirB5, VirB6, VirD4, etc.) for the transfer of the T-DNA (Tra and trb genes), and (iii) T-DNA integration (VirC2, VirE2, VirE3, VirD5, etc.) into the plant genome (Table 2A).

The VirA and VirG protein families involved in host recognition were detected in all but CG8 (no VirA) de novo sequenced virulent and nonvirulent genomes as well as the 3 reference genomes. For *A. fabrum* C58, it is known that the ubiquitously expressed 2-way component phospho-relay system VirA/VirG detects and transduces signals of wounded plants and the phosphorylated VirG activates transcription of the Vir operon (Brencic and Winans 2005; Wise and Binns 2015). Nevertheless, VirG belongs to a large family of positive regulators responding to external phenolics, monosaccharides, and pH (Winans et al. 1986), indicating that the predicted protein family plays multiple roles besides virulence regulation.

The agrobacterial VirB/VirD4 system forms the T4SS, dedicated to deliver the T-DNA and effector proteins into plant cells in the course of infection. Most protein homologs of the T4SS encoded on the 3 reference Ti plasmids (pTiC58, pTiS4, pTiK309, pTiAg57) were present in the virulent *A. vitis* isolates (CG1 to CG3). However, our virulent isolates harbor most likely a T4SS variant distinct from the one of the reference Ti plasmids because sequences encoding VirB7, VirB8, and VirB9 were not present in the genome data of CG1 to CG3. These Vir proteins have stabilizing functions and are either located in or at the outer (VirB7, VirB9) as well as inner (VirB8) bacterial membrane (Gordon and Christie 2014).

The agrobacterial factors translocated into host cells by the T4SS are essential for T-DNA integration and include the VirD2-T-DNA complex, VirD5, VirE2, VirE3, and VirF (Schrammeijer et al. 2003; Vergunst et al 2005). These protein families have different functions and were present in the genomes of our virulent isolates CG1 to CG3. Taken together, the genome sequences of our *A. vitis* isolates CG1 to CG3 harbor the genetic repertoire required to cause CG disease (supplementary fig. S1, Supplementary Material online).

### All *Rhizobiaceae* Isolates Harbor the Machinery for Ti Plasmid Conjugation

CGs are a perfect niche for plasmid conjugation within a bacterial population (Dessaux and Faure 2018). The proteins necessary for replication (RepABC) and transfer (Tra, Trb) of Ti plasmids have been previously described in detail (Lang and Faure 2014). Representatives of these protein families existed in our virulent and nonvirulent *Rhizobiaceae* genomes (Table 2B) but not in the isolates CG7 (*Pseudomonas* sp.) and CG8 (*Rahnella* sp.) that were also part of the CG microbiota (Faist et al. 2016). The genomes of the 3 virulent *A. vitis* isolates (CG1 to CG3) each contained a region (Fig. 3B to D, black arrow heads) coding for pTi replication (yellow bars) and transfer (orange bars) which had highest homology to pTiAg57 of *A. fabrum* Ag57 (≤100%, green connecting lines), less homology to pTiC58 of *A. fabrum* C58 and pTi309 of *A. vitis* NCPPB3554 (≤90%, pale green connecting lines), and least to pTiS4 of *A. ampelinum* (≤80%, orange connecting lines). Among the nonvirulent *Rhizobiaceae* isolates, CG4 (*A. divergens*) and CG5 (*Rhizobium* sp.) seem to lack the sequence encoding replicase RepC based on the ortholog prediction (Table 2). However, the automatic gene annotation by the National Center for Biotechnology Information (NCBI) revealed multiple genes classified as repC. This indicates the limitation of our gene assignment method, which can lead to false negatives. The also nonvirulent *Rhizobiaceae* isolate CG6 (*A. rosae*) contained an almost identical sequence region to the virulent isolate *A. vitis* CG1 (Fig. 6A, green connecting line) involved in plasmid replication (yellow bars) and transfer (orange bars) suggesting the presence of a plasmid but not of a virulent Ti plasmid. Regions involved in virulence (green bars) and T-DNA transfer (red bars) found in the genomes of C58 and CG1 were lacking in CG6.

*Quorum sensing* and *quorum quenching* regulate conjugation of Ti plasmids between bacteria and horizontal transfer of the T-DNA into the host genome (Dessaux and Faure 2018). Opines synthesized by T-DNA transformed tumor cells play a key role in bacterial conjugation since they activate the conjugal transfer (*tra*) genes (Mel and Mekalanos 1996). Sequences of the predicted protein families involved in *quorum sensing* (TraI, TraM TraR) were present in all virulent and nonvirulent *Rhizobiaceae* genomes (Table 2B) but not in *Pseudomonas* sp. (CG7) and *Rahnella* sp. (CG8). The fact that similar *quorum sensing* genes existed in the virulent and nonvirulent *Rhizobiaceae* isolates raised the possibility of cross species communication. This seems likely between our virulent *A. vitis* (CG2) and nonvirulent *Pseudomonas* (CG7) isolates as well as between *A. vitis* (CG3) and *Rahnella* (CG8) because these resided in the same tumors B and C, respectively (supplementary table S3, Supplementary Material online). Representatives of the lactonase protein families (AttM/BlcC, AiiB) which degrade the bacterial *quorum sensing* signal molecule N-acyl homoserine lactone and thus are functioning in *quorum quenching* were found in the virulent *A. vitis* (CG1), the nonvirulent *A. rosae* (CG6), and *Pseudomonas* (CG7; Table 2C). *Quorum quenching* reduces the Ti plasmid transfer frequency and thereby the host transformation events by virulent agrobacteria (Haudecoeur et al. 2009; Haudecoeur and Faure 2010; Lang et al. 2016). Since *A. vitis* CG1, *A. rosae* CG6, and *Pseudomonas* CG7 were from different CGs, they cannot negatively influence plasmid conjugation among each other or between *Rahnella* (CG8) and *A. vitis* (CG3) and *Pseudomonas* (CG7) and *A. vitis* (CG2). Thus, the latter 2 isolate pairs of tumors B and C each have the genetic repertoire to communicate via *quorum sensing* signals and transfer genetic material.

### Opine Utilization Plays a Key Role in CG Colonization

The possibility for nonvirulent bacteria to acquire the ability for opine utilization is advantageous for them in the CG environment. An overview of the genes involved in opine utilization by agrobacteria is provided in Vladimirov et al. (2015) and Table 2C. In CGs, different opines can coexist (Petit et al. 1983), and their recognition is conferred by various periplasmic binding proteins (PBPs). The PBPs OccT and NocT can recognize nopaline and octopine, respectively, or in the case of NocT even both opines (Vigouroux et al. 2017). Association of PBPs with ATP binding cassette transporters (OocQ, NocQ, OocM, NocM, OccP, NocP) enables the import of opines into bacterial cells (Zanker et al. 1992). In our study, NocT and OccT homologs were present in all CG-associated genomes and representatives of the ABC transporters (OccPch, NocP1 and OccQe, OccMch, etc.) even in high copy numbers (Table 2C). A similar ubiquitous occurrence showed protein families for opine sensing (NocR, AccR). These function in transcriptional activation of opine uptake and catabolism genes (Subramoni et al. 2014). Hence, all CG-associated bacteria harbor the genetic potential for opine uptake and sensing.

Ti plasmids carry the genes for opine synthesis (*nos*, *ocs*, *vis*, cus, acs, etc.) by plant cells as well as the corresponding catabolism genes (*noxA*/*noxB*, *ooxA*/*ooxB*, rocF, etc.). The octopine synthase gene sequence (*ocs*) is similar to the one of vitopine synthase (*vis*) and therefore joins the same protein family, while the nopaline synthase gene (*nos*) forms a separate family (Canaday et al. 1992). We found homologs of the opine synthases Ocs, Vis, Cus, and Acs/AcsX but not Nos, Nos-like, and Mas1 in the genomes of the virulent isolates GC1 to GC3 only (Table 2C). Since no Nos/Nos-like homologs were found in the genomes of isolates CG1 to CG3 and these metabolized octopine much better than nopaline (supplementary table S3, Supplementary Material online), we suggest that our 3 virulent *A. vitis* isolates harbor an octopine/vitopine type Ti plasmid rather than a nopaline type.

In contrast to anabolism, which was restricted to the virulent isolates, all analyzed CG-associated bacterial genomes harbored sequences encoding enzymes for opine catabolism (Table 2C). The protein families of OoxA/NoxA and OoxB/NoxB were found in all genomes, except of the isolate CG4 (*A. divergens*) which lacked OoxB/NoxB. OoxA/NoxA together with OoxB/NoxB are 2 soluble polypeptides, which are both required for the first step of opine utilization (Zanker, et al. 1994) and may explain why CG4 could not metabolize nopaline or octopine (supplementary table S3, Supplementary Material online). Furthermore, the arrangement of the genes encoding the proteins for opine catabolism is essential for an effective function (Zanker, et al. 1994). The order and orientation of the genes involved in opine sensing (*nocR*), uptake (*occQe*, *occPch*, *nocP*), and catabolism (*ooxB*, *rocF*, *ocd*) in the vicinity of *ooxB/ooxA* of the type IVb reference Ti plasmid of *A. vitis* NCPPB3554 (Fig. 5) was the same in our virulent isolates (CG1 to CG3). It differed in the genomes of the nonvirulent isolates CG4, CG5, CG7, and CG8, but not CG6. *Rhizobiaceae* sp. CG5 metabolized octopine well and harbored the essential catabolism genes *ooxA* and *ooxB* in the correct order and orientation but the other genes in some distance. The gene order and orientation in the genome of *A. rosae* CG6 was the same as in the virulent isolates. This points to a different event of acquisition for the opine transport and catabolism genes and might have an impact on opine utilization which was less effective by CG6 compared with the virulent isolates CG1 to CG3. The sequences for opine transport and catabolism of CG6 revealed a high degree of similarity to the pAtK84b plasmid of the strain *A. rhizogenes* K84 and an even higher to the pAtAg67 plasmid from *A. fabrum* Ag57 (Fig. 6B, gray bars). *Agrobacterium rhizogenes* K84 harbors a nopaline-catabolic plasmid (pAtK84b) and *A. fabrum* Ag57 an octopine/cucumopine-catabolic plasmid (pAtAg67; Hooykaas et al. 2022) which lack the Vir and T-DNA regions like the genome of CG6. *Agrobacterium rhizogenes* K84 is frequently used as biocontrol agents to prevent CG development (Clare et al. 1990). Thus, OC plasmids have an ecological impact on controlling CG disease severity, possibly via advantages in the competition for opines with virulent strains and the secretion of antimicrobial substances (Platt et al. 2014).

The *Pseudomonas* isolate CG7 contained 2 regions which showed the essential succession of *ooxA/noxA* and *ooxB/noxB* but lacked sequences for *arc*, *odh*, and *ocd/rolD* (Fig. 5). Nevertheless, the in vitro growth assay confirmed utilization of octopine by *Pseudomonas* CG7. Previously, it was shown that in CGs of grapevines, bacteria other than *A. vitis* can utilize opines, including *Pseudomonas* strains (Nautiyal and Dion 1990; Canfield and Moore 1991; Moore et al. 1997; Wetzel et al. 2014; Eng et al. 2015). However, the molecular mechanism behind it is yet unknown. The *Rahnella* isolate CG8 harbored the essential genes for opine catabolism, but those for opine transport were located downstream of *ooxA/noxA* and *ooxB/noxB* (Fig. 5). Therefore, one might speculate that the reversed order of the essential opine-catabolic and uptake genes may prevent opine utilization by Rahnella (CG8). The reference genome of *Rahnella aquatilis* HX2 (No. 34 in Table 1) does also not harbor the essential genes for opine utilization and was proposed to function as biocontrol in confining CG disease (Chen et al. 2007). Consequently, the nonvirulent CG isolates *Rhizobiaceae* sp. CG6, *Pseudomonas* CG7, and *Rahnella* CG8 have the potential to control CG disease.

In root nodules, acquisition events have previously been suggested between alpha-, beta-, and gammaproteobacteria (Shiraishi et al. 2010; De Meyer et al. 2016; Ryu et al. 2020). Thus, an exchange of gene sequences between our alphaproteobacterium *A. vitis* (CG1, CG2, CG3), betaproteobacterium *Rahnella* (CG8), and the gammaproteobacterium *Pseudomonas* (CG7) seems possible in CGs since CG2 and CG7 as well as CG3 and CG8 resided in the same CG. This might equip the nonvirulent members of the microbial CG community with the machinery to utilize opines. Moreover, the transfer of the opine utilization machinery to beneficial plant bacteria could stabilize their population in an opine-enriched environment. Taken together, in CGs, virulent *Rhizobiaceae* provide an opine-rich niche for themselves and other opine-catabolizing bacteria as well.

## Conclusion

On a genetic level, gene duplication, rearrangements, and interspecies horizontal gene transfer may be important for the dissemination of opine utilization among the CG-associated bacterial community. Our results highlight the distribution of sequences encoding proteins for opine catabolism (utilization of the CG-specific nutrient), but not for virulence (induction of the CG disease) among the bacterial community of the CG. The extent of CG development correlates with the number of transformation events and plant vigor. Grapevines developing small CGs show no growth limitations (Schroth et al. 1988; Ferreira et al. 1992), and the grapevines of this study did also not display an obvious phenotype. Opines are unique compounds produced by CGs which can only be metabolized by bacteria-expressing enzymes for opine uptake and degradation. In contrast, other plant nutrients must be shared by the whole CG community. Competition for opines between virulent *A. vitis* and nonvirulent bacteria could balance the composition of the microbial community in a CG and, thus, promote or limit the degree of the CG disease. Therefore, a better understanding of the factors balancing the composition of the microbial community and providing a bacterial community, which is dominated by beneficial microbes, might lead to novel disease management strategies.

## Materials and Methods

### Isolation and Cultivation of Bacteria

In 2011, 2012, and 2013, we isolated bacteria from CG material sampled in vineyards of Franconia, Germany. The grapevine cultivars consisted of the scion Cabernet Dorsa, Scheurebe, and Müller Thurgau grafted onto the rootstocks 5BB and SO4 (NCBI, BioProject: PRJNA624984). CG material was ground (2 min, 30 Hz) using a ball mill (Retsch, Hannover, Germany), and 300 mg CG powder was suspended in super purified water (RotisolV high-performance liquid chromatography [HPLC] gradient grade; Roth). After incubation for 2 h at 28 °C, the supernatant was used to create serial dilutions at a ratio of 1:10. Agar plates containing yeast extract broth (YEB; 0.5% [wt/vol] tryptone, 0.5% [wt/vol] yeast extract, 0.5% [wt/vol] sucrose, 1.23% [wt/vol] MgSO_4_ [AppliChem, Darmstadt, Germany], 1.5% [wt/vol] Agar-Agar Kobe I [Carl Roth, Karlsruhe, Germany]) supplemented with 213 µM cycloheximide (CHX; Sigma-Aldrich, St. Louis, MO, USA) were used for bacterial growth at 28 °C. By growing colonies on rifampicin-containing yeast extract agar (RIF-YEA, 10 µg/mL) plates, spontaneous rifampicin-resistant derivatives were selected for tracking them in their natural environment. Single colonies were subcultured at least 5 times on YEA-CHX-RIF plates and used for de novo shotgun sequencing.

### Virulence and Opine Growth Assays

Virulence assays were performed by inoculating bacterial suspensions into *Vitis vinifera* stems as described by Faist et al. (2016), stems of 4-wk-old *A. thaliana* (accession Col-0), and *N. benthamiana* plants according to Gohlke et al. (2013). Pictures were taken using a charge-coupled device (CCD) camera (Leica DFC500, Leica Microsystems GmbH) attached to a stereo microscope (Leica MZFLIII, Leica Microsystems GmbH). Opine utilization assays were performed in liquid AB minimal medium (K_2_HPO_4_ 3 g/L; NaH_2_PO_4_ 1 g/L; MgSO_4_-7H_2_O 0.3 g/L; KCl 0.15 g/L; CaCl_2_ 0.01 g/L; FeSO_4_-7H_2_O 2.5 mg/L; pH 7) supplemented with 1 mg/mL octopine or nopaline (Vigouroux et al. 2017) or with sucrose + NH_4_ and glycerol as controls. Bacterial growth was determined as optical density (OD_600_) and defined as follows: no growth, OD < 0.1; very weak growth, 0.1 ≤ OD < 0.2; weak growth, 0.2 ≤ OD < 0.5; and growth, OD ≥ 0.5. The growth experiments with opines were repeated 5 times and the control experiments 2 (glycerol) to 3 (sucrose + NH_4_) times.

### Sequencing and Identification of Bacterial Genomes

Eight bacterial CG isolates (CG1 to CG8) were sequenced either using an Illumina MiSeq (2014, CG1 to CG2 and CG4 to CG6, 2 × 250 bp V2 chemistry) or a NextSeq (2017, CG3 and CG7 to CG8, 2 × 150 bp mid-throughput v2 500/550 kit) after library preparation with the Nextera XT and 24 index kits. Raw reads were quality filtered, corrected (Q30), and assembled using SPAdes 3.10.1 (Bankevich et al. 2012). The assembled sequences were screened for bacterial contaminations using BlobTools (Laetsch and Blaxter 2017) with taxonomic assignment via BLAST (Altschul et al. 1990). Contigs are assembled DNA sequences and in this study synonyms of scaffolds and nodes. On the contigs, rRNAs, tRNAs, genes (filtered open reading frames), and CDS were annotated with Prokka v1.12 (Seemann 2014) and Barrnap 0.9 (Torsten Seeman, https://github.com/tseemann/barrnap). Completeness of the genomes is indicated by the abundance of the 107 essential genes (Dupont et al. 2012). The overall genome coverage was calculated by mapping the original reads back onto the assembled contigs using Bowtie2 (Langmead and Salzberg 2012). The full-length 16S rRNA gene sequences of the de novo draft genomes were taxonomically identified with the EZBioCloud database (Yoon et al. 2017). These 16S sequences were also matched with BLAST against the data set of 16S v4 amplicon sequence variants (ASVs) by Faist et al. (2016) to account for their relative abundances in the whole bacterial community. We calculated a phylogenetic tree using bcgTree (Ankenbrand and Keller 2016), including our *Rhizobiaceae* isolates as well as 94 randomly selected *Rhizobiaceae* bacteria and 4 *Bradyrhizobium* genomes as an outgroup from EZBioCloud. The accession numbers for the raw reads at NCBI are JABAED000000000, JABAEE000000000, JABAEF000000000, JABAEG000000000, JABAEH000000000, JABAEI000000000, JABAEJ000000000, JABAED000000000, and JABAIN000000000 while for the annotated assemblies, it is DOI: 10.5281/zenodo.3752520.

### Comparative Genomics

A total of 34 reference genomes were used in addition to the 8 genomes from this study. The references include 14 strains of *A. vitis*, 2 strains of *A. fabrum/tumefaciens*, 8 strains of *A. rhizogenes*, 6 strains from the genus *Pseudomonas*, 1 from *Rahnella*, 1 from *Sphingomonas*, and 2 *Curtobacterium* strains. Strain details and accession numbers are listed in Table 1 and supplementary table S1, Supplementary Material online. Most of the *A. tumefaciens*, *A. vitis*, and *A. rhizogenes* references are described in Weisberg et al. (2020) including a classification of their oncogenic plasmid and opine metabolism capabilities. OGs based on amino acid sequences of our isolates (CG1 to CG8) and the reference genomes were identified by OrthoFinder (Emms and Kelly 2015). Gene names were transferred from the reference genomes to all genes of the cluster. Protein families including known Ti plasmid proteins from the references were sorted into the following functional groups: (i) virulence, (ii) plasmid replication and transfer, and (iii) utilization of opines (Table 2). DNA sequences were aligned with lastZ (Harris 2007) and visualized with AliTV (Ankenbrand et al. 2017). For this comparison, 2 additional, recently described plasmids were included, namely pTiAg57 and pAtAg67 (Hooykaas et al. 2022). Genes of these 2 plasmids were assigned to orthogroups by best BLAST hit (Altschul et al. 1990) with e-value below 10^−12^. For an unsupervised approach, shared and unique OGs of different bacterial species were displayed in UpSetR (Conway et al. 2017). For identification of putative opine clusters, we analyzed the genomic neighborhood of regions, where homologs of both OoxA/NoxA/VoxA and OoxB/NoxB/VoxB genes are found. We selected fragments of 15 kbp downstream and upstream of these genes for direct comparisons. If not indicated otherwise, gene functional descriptions are from the STRING database (https://string-db.org/, March 2019).

## Supplementary Material

evad228_Supplementary_Data

## Data Availability

The genome sequences obtained in this study have been deposited at NCBI with BioProject accession number PRJNA624984, and annotated assemblies are available from Zenodo with doi:10.5281/zenodo.3752520.
