# Supplementary material for: Opportunistic Bacteria of Grapevine Crown Galls Are Equipped with the Genomic Repertoire for Opine Utilization
Source: Genome Biol Evol. 2023 Dec 12;15(12):evad228. doi: 10.1093/gbe/evad228 (PMC10745273; doi:10.1093/gbe/evad228)
Supplement: evad228_Supplementary_Data [file evad228_supplementary_data.pdf]

Supplementary Material

Supplementary Fig. 1

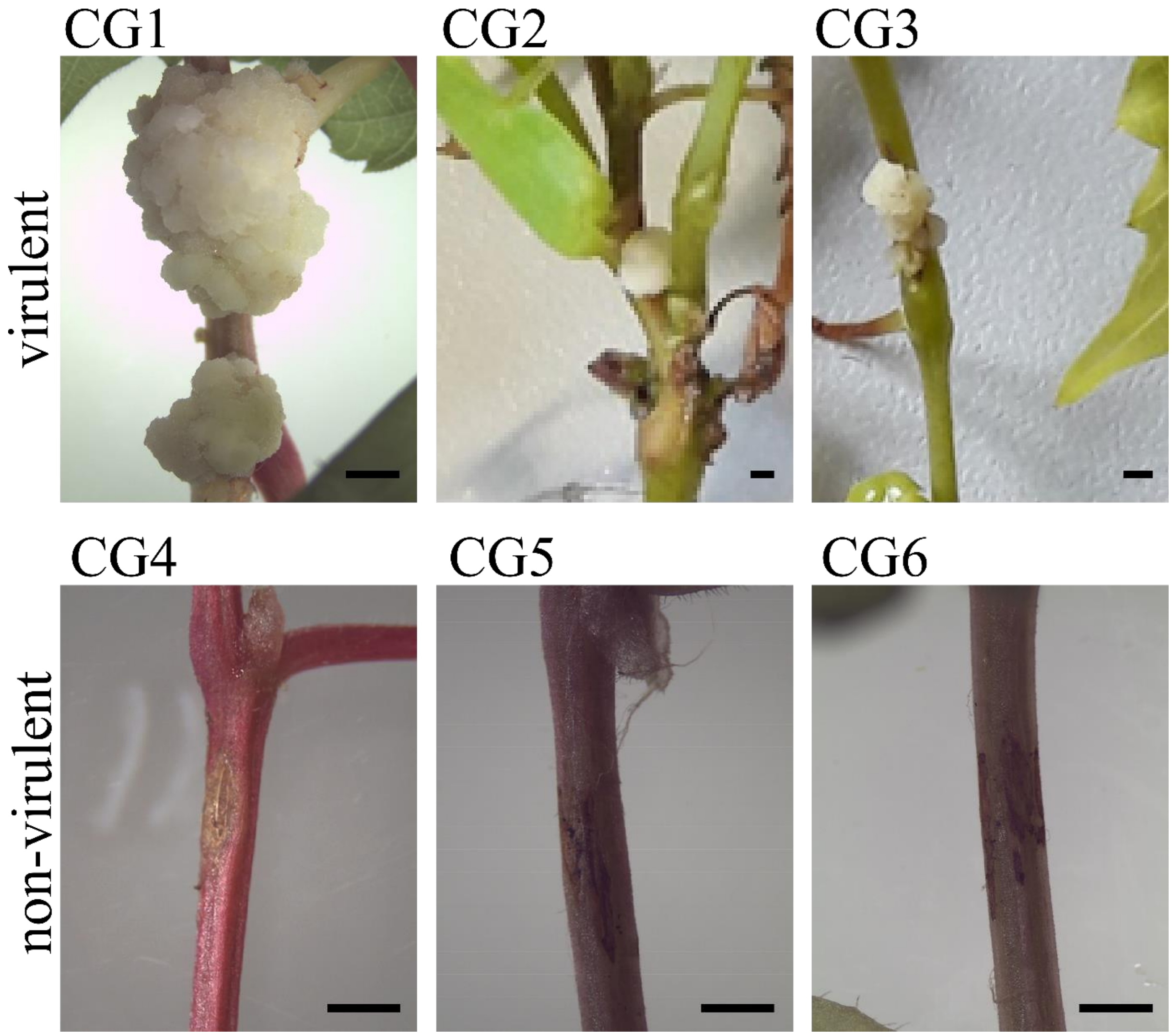

Supplementary Fig. 1. Virulence assay with the bacterial isolates CG1-CG6 and grapevine seedlings. Upper panel: Inoculation of the virulent isolates (CG1-CG3) caused crown gall formation in grapevine stems. Lower panel: Inoculation of the non-virulent isolates (CG4-CG6) induced no crown gall development at the wounded areas of grapevine stems (arrows). Bars represent 0.5 cm.

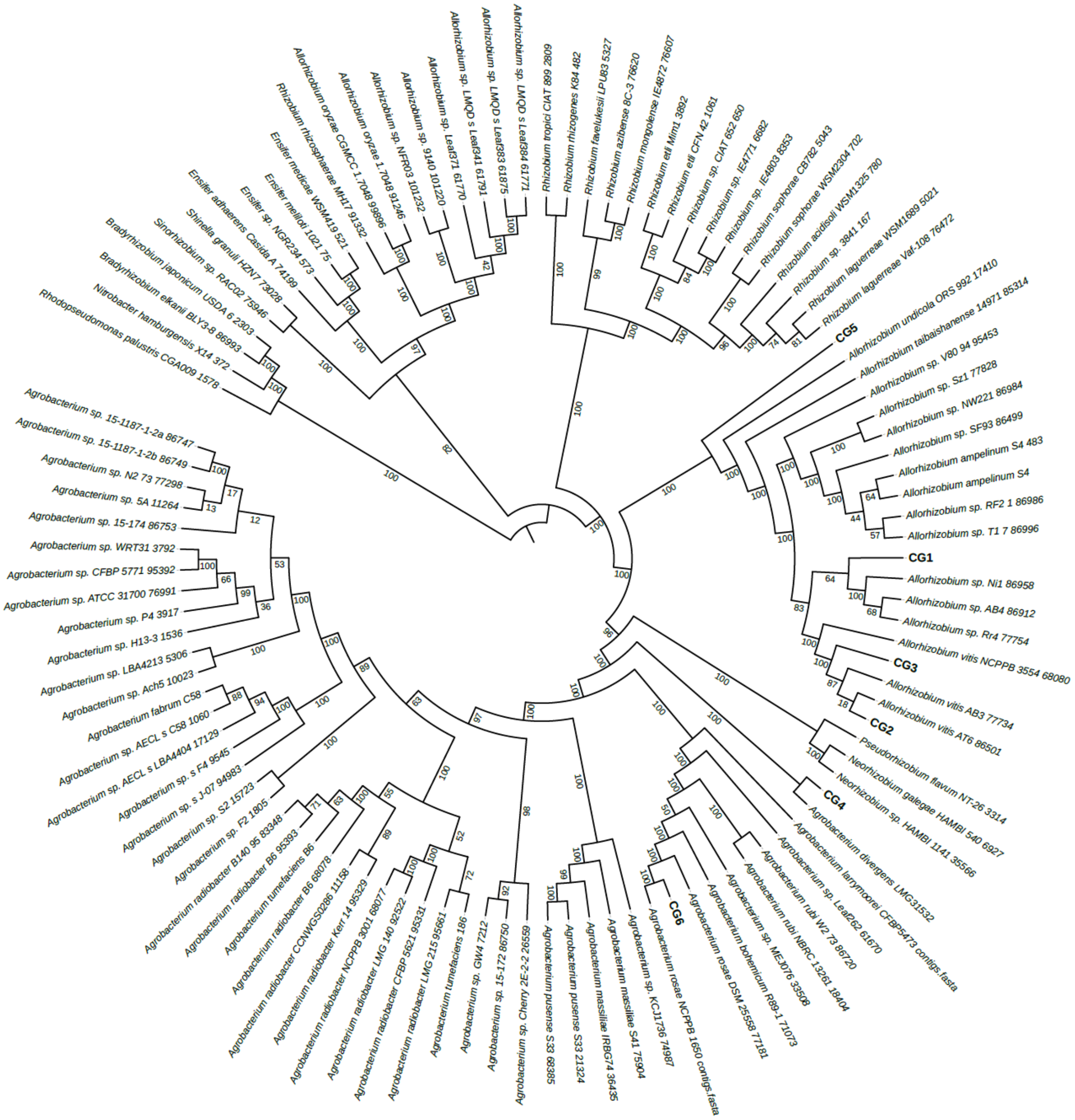

Supplementary Fig. 2

Supplementary Fig. 2. Phylogenetic tree based on 107 single copy housekeeping genes of members of the Rhizobiaceae family. The *de novo* sequenced isolates CG1-CG6 are integrated into a phylogenetic tree of Rhizobiaceae reference genomes from the EZBioCloud database.

Supplementary Table S1. Information on bacterial isolates of grapevine crown galls sampled from different vineyards in the Franconian region, Germany. Listed are 1. the characteristics of the isolates and 2. the relative abundance of 16S rRNA V4 amplicons in crown galls (% CG) and non-galled graft unions (% NG; Faist, et al 2017). Significant differences (p-value ≤ 0.05) between the graft unions with and without a crown gall were calculated according to one-way ANOVA followed by post hoc Tukey analysis for multiple testing.

**1. Characteristics**					**2. 16S rRNA V4**		
**Isolate**	**Tumor ID**	**Sampling date, vineyard**	**Reference strain**	**%**	**CG %**	**NG %**	**P-value**
CG1, *Allorhizobium vitis*	**A**	Jan-09-2011, Himmelstadt, Germany	*Allorhizobium vitis*, NCPPB3554(T)	99.9	11	0.3	<0.0001
CG2, *Allorhizobium vitis*	**B**	Jul-17-2013, Ravensburg, Germany	*Allorhizobium vitis*, NCPPB3554(T)	99.9	11	0.3	<0.0001
CG3, *Allorhizobium vitis*	**C**	Oct-30-2013, Himmelstadt, Germany	*Allorhizobium vitis*, NCPPB3554(T)	99.9	11	0.3	<0.0001
CG4, *Agrobacterium divergens*	**B**	July-17-2013, Ravensburg, Germany	*Rhizobium* sp., H13-3	98.8	ND	ND	ND
CG5, *Rhizobiacea sp.*	**D**	Oct-24-2012, Sommerhäuser Reifenstein, Germany	*Rhizobium* sp., H13-3	98.6	0.8	0.8	0.9
CG6, *Agrobacterium rosae*	**E**	Oct-30-2013, Himmelstadt, Germany	*Rhizobium* sp., Ch11(T)	99.7	1.5	4.5	0.2
CG7, *Pseudomonas*	**B**	July-17-2013, Ravenburg, Germany	*Pseudomonas* sp., DSM 13194(T)	99.9	15.6	0.5	<0.0001
CG8, *Rahnella*	**C**	Oct-30-2013, Himmelstadt, Germany	*Rahnella* sp., Y9602	99.9	5.5	0.4	<0.1

Supplementary Table S2. Features of the assembled draft bacterial genomes (CG1-CG8). N50 and N90 indexes list the length of the smallest contig that build 50% and 90%, respectively of the draft genomes. cont, contig.

**Draft genome**	**Total cont**	**Cont >1kb**	**20 longest cont [Mb]**	**Total size [Mb]**	**N50 [kb]**	**N90 [kb]**	**GC [%]**	**Largest Kmer-coverage**	**Overall alignment rate [%]**
A. vitis CG1	427	58	5.75	6.31	457	80.5	57.4	45.7	98.53
A. vitis CG2	176	39	5.29	5.50	599	108	57.7	34.0	98.78
A. vitis CG3	212	149	2.90	6.10	89.7	24.6	57.7	14.3	86.86
A. divergens CG4	117	23	5.66	5.71	458	159	55.0	28.2	98.64
Rhizobiacea sp. CG5	223	54	5.39	6.58	262	70.2	61.5	21.5	98.74
A rosaeCG6	148	26	5.68	5.74	472	204	56.6	49.4	97.99
Pseudomonas CG7	392	182	2.48	6.87	65.5	17.6	60.7	19.6	84.98
Rahnella CG8	175	73	4.07	5.63	186	43.0	52.3	43.5	87.92

Supplementary Table S3. Bacterial growth assays in liquid medium with AB salts and supplemented with either nopaline, octopine, glycerol, or sucrose+NH_4^+ as sole C and N source. Optical density at 600 nm (OD_{600}) was measured after 48 h. Mean values of OD_{600} represent 5 replicates of two experiments. As control served the *Agrobacterium* strains C58 utilising nopaline and B6 octopine. OD < 0.1, no growth; 0.1 ≤ OD < 0.2, very weak growth; 0.2 ≤ OD < 0.5, weak growth; OD ≥ 0.5, growth. Red and blue colours indicate presence of isolates in the same tumor.

Tumor			Nopaline	Octopine	Glycerol	Sucrose+NH_4^+
**A**	***Allorhizobium vitis***	**CG1**	0.37 +/- 0.12	0.58 +/- 0.16	1.42 +/- 0.17	0.85 +/- 0.00
**B**		**CG2**	0.14 +/- 0.02	0.87 +/- 0.11	1.97 +/- 0.06	3.40 +/- 0.42
**C**		**CG3**	0.26 +/- 0.07	1.14 +/- 0.42	1.98 +/- 0.03	2.10 +/- 0.07
**B**	***Agrobacterium divergens***	**CG4**	0.10 +/- 0.03	0.14 +/- 0.07	1.85 +/- 0.15	0.10 +/- 0.07
**D**	***Rhizobiaceae sp.***	**CG5**	0.15 +/- 0.03	1.01 +/- 0.25	1.92 +/- 0.04	2.68 +/- 0.04
**E**	***Agrobacterium rosae***	**CG6**	0.12 +/- 0.05	0.31 +/- 0.12	0.43 +/- 0.15	0.25 +/- 0.00
**B**	***Pseudomonas***	**CG7**	0.14 +/- 0.02	1.10 +/- 0.17	1.97 +/- 0.06	3.50 +/- 0.35
**C**	***Rahnella***	**CG8**	0.16 +/- 0.22	0.08 +/- 0.04	1.97 +/- 0.06	3.58 +/- 0.25
**-**	***Agrobacterium tumefaciens***	**B6**	0.14 +/- 0.05	0.71 +/- 0.16	1,56 +/- 0.02	2.45 +/- 0.14
**-**		**C58**	1.33 +/- 0.70	0.06 +/- 0.05	1.99 +/- 0.02	2.93 +/- 0.04
**-**	**non-inoculated**	***ni***	0.00 +/- 0.00	0.00 +/- 0.00	0.00 +/- 0.00	0.00 +/- 0.00
